# Narrative Inquiry: An Interview With Michael Bamberg

**DOI:** 10.5964/ejop.v12i1.1128

**Published:** 2016-02-29

**Authors:** Michael Bamberg, Carolin Demuth

**Affiliations:** a Clark University, Department of Psychology, Worcester, MA, USA; b Center for Developmental and Applied Psychological Science, Aalborg University, Aalborg, Denmark

**Carolin Demuth:** Michael, your work over the past decades has contributed significantly to our understanding of identity, particularly, narrative identity. Can you talk a little bit about the story of how you first got interested in the topic?

**Michael Bamberg:** This is typically the question where people tell a self-narrative of a transformative experience; but, you know, I approach this question also, “what am I doing here?” in identity research, from a theoretical perspective, and my entry point to narrative studies, narrative inquiry, and also identity research, is coming in as a developmental theorist. Often people assume that if they study a population like children, or infants, or emerging adulthood, they do developmental research. But, in reality I think, what they do, is they study a population, typically also, a population that is still in the process of developing, or becoming or changing, and the assumption there is that as adults, we have emerged and then, there is no more development. But that is, I think, a misconception. It’s also highly problematic, because, what really is development? And this question I would tackle with the assumption that there is change going on in everyday encounters, in mundane situations, throughout the *whole* life.

Although this resembles a little bit lifespan psychology, I am entering this from a different angle, and that is that the changes that are taking place are *micro* changes, are changes that are usually not the *big* kinds of, which are, typically retrospective reflections, where we think we have changed from one person to another. But there are micro processes, within which change typically takes place. And this is exactly where change and consistency are in cahoots, or working together. So [I am] looking at the micro changes, within the context of what is called socio-changes or socio-genesis, and ontogenesis, where a person comes into being, and where a person also becomes a social being; in this interplay, these micro processes that are taking place, constitute, if you want to call it, change. They are the territory, the landscape within which development is forming, and within which then it becomes a territory for inquiry. So these are the kinds of developmental processes, I think each developmentalist is interested in. And that is where the issue of identity and identity formation, these types of changes that are taking place, are at the core of psychology; but not just psychology, also of a number of fields within sociology and anthropology and possibly we will get into this later. But there is this *balance* or *navigation* of what remains *constant*, and what *changes*. I think that is at the core of developmental studies, or developmental perspective, and this also at the same time is at the core of what we can call identity formation, and also then the sense of self that is emerging in these formative processes. So that will be my answer to the question “How do I enter identity and identity studies”.

**Carolin Demuth:** So what is it that is so special about self-narrative that makes it crucial for identity?

**Michael Bamberg:** My orientation point with regard to narratives is that self-narratives are probably nothing special. They *are* to a lot of narrative psychologists, and they are also to traditional psychologists who are not really using story-telling or narratives as a means to get into issues of identity or sense of self. I think behind that, that self-narratives are the most relevant way of getting into identity analysis, are the moments of self-reference, where we so to speak step outside of our bodies and look at ourselves and refer to ourselves. So that’s where these moments of self-reflection for traditional narrative psychologists become the center for identity and identity analysis. And I’m worried about this, because this assumes that somewhere somehow in our social development en route to modernity we have learned this process of stepping outside of ourselves and reflect on ourselves, and typically through institutions like the confession – Saint Augustine I think is a landmark here in this development, but then to modernity in the last two, three hundred years, where we have become more self-oriented, self-centered as well – not in a typically negative sense, but in terms of this socio-genesis, that we have become more of a society of individuals. And within this, of course, there is this ability to step outside and look at ourselves from a different angle, and reflect on ourselves. And at the high point of this socio-genesis is the turn to a therapeutic orientation where within the last hundreds years, we have become more and more, we can call it, *aware,* but also possibly obsessed with this notion that we can dig into this self as something that has an interior with something like a self memory, and then we can work with this in order to find out who we really are. But again, this is, I think a very traditional view and this view has a lot of problems.

Let me just cut through this where I would like to enter: the role of narratives is less in *self*-narratives – they *can* be self-narratives, they *can* thematize myself – but in *any* kind of narrative practice, in story-telling practices so to speak, we *practice* a sense of who we are, we practice first of all, to put things into a temporal continuity and discontinuity. So there is a temporal aspect of mapping out characters that *can* be the self, but it doesn’t *have* to be. Typically, we design characters who have a bit of a history coming to a present and possibly even launching into something that follows next as a future. So in this we are laying out characters, but at the same time we view these characters from a particular perspective, we view them as navigating a sense of *constancy* and *change*. Typically there is some character *development*, in particular in modernity in the novel, the character *becomes* him or herself, so there is a change over time.

[Contrib contnr1]But then we also lay these characters out in terms of “how is this character different from others?” in stories, in particular how is the character a protagonist versus antagonist. But then there is also a valuing of these characters, in terms of how they *differ,* [e.g.] in terms of gender. If you want to tell a story that has the romantic plot, then traditionally, typically, it is a hetero story – not anymore, it doesn’t have to be – but it is typically the story where males are characterized, drawn out, differently from females. And these differences are brought to the fore, and in a way embraced and celebrated. Of course there’s a change, in particular recently, in how these plots are formed and the roles these characters are playing, so to speak. So the characters are designed in terms of *differences* and *sameness*, different in terms of their ethnicity, of their gender, their age, local, regional background [etc.]. And they are also drawn out in terms of similarities, in-group characteristics, if you want to call it.

And the third territory here, along which characters are designed is in terms of *agency*: how agentive are they characterized or how passive, in what kinds of passive roles are they placed so to speak. If you want to draw out a hero, then this hero is kind of almost *planning* what he or she – typically *he* in the tradition of heroic actions – is doing next: the plan, the intention is there, and then the intention leads to actions and changes in the world. So, the characters are drawn up highly transitive, in that sense. In the victim story or in a suffering story, we draw out the characters typically in an *in*agenative way as somebody who is the victim of typically the actions of others, but it can also just be happenings or bad luck or fate, thunder storms and snow, where these consolations are put in a way that the character is placed into conditions, as a recipient. I also call these three territories *dilemmas* because the speaker has choices to place these characters into these three territories or arenas or spaces in different ways. This of course also happens when we talk about *ourselves* but I think that is a variant of *general* story-telling, and it might be a special variant, but I think nothing particular is happening here that is not already visible and done in story-telling about *others*, and also in fictional story-telling.

But what comes in here, and I really would draw up an important difference between identity researchers who put all their money on *self* reference and on stories where people *reflect* on themselves – is that in *all* story-telling, there is a *practice* in story-telling, and we relate not only with words but also with our bodies to our audiences; and I think, this is where gestures, where facial expressions, where gaze become relevant in order to package – package is possibly the wrong expression but – there is a unit of how, whatever is said is expressed bodily and whatever is said, is said verbally. This unit of verbal and bodily expression in story-telling is something that is making a difference – and also that is analyzable, and in particular now that we have visual means where we also can go into the visual means of story-telling and bring this into the package that we can use when we analyze and work with stories analytically.

[Contrib contnr2]**Carolin Demuth:** This is basically also your critique of the big story approach of life stories, where people reflect on their lives. You make a strong point in saying that identity is achieved in situated practices in everyday mundane interactions, through small stories, fragmented stories. But don’t you think that *big* life stories still have relevance for identity research?

**Michael Bamberg:** This brings up the issue that – about 10 years ago, I think we started to tackle this difference between big stories and small stories or narrative practices. Let me just clarify this a little bit: the *small story approach*, or the *narrative practice approach*, assumes that stories typically occur in mundane everyday situations. When we interact, when we talk, that’s where we *at times*, not all the time, we launch into story-telling mode or we break into story-telling mode. And we *mark* this also: ‘Oh I remember’ or ‘Oh that reminds me’ or ‘Let me tell you the story of what…’, but *not all the* time do we mark it; sometimes we just launch into them, but it is very typical. I mean if you look at the bodily reactions also that we have on video tape, even in 10 year olds, when somebody is taking the floor with a small story, the others lean back and then towards the end, again move forward – and this happens already, two, three, four seconds before the story teller ends the story – the others move in and there is a signaling, a bodily signaling, that ‘I’m done with my story, the floor is open and I want some reactions to the story’. So these are the kinds of *situated interactive contexts* within which stories have their function. And they do, with regard to identity research now, they do *identity work*; and we can go into this in a little bit more detail.

But now, you asked, isn’t there something that big stories also accomplish, also do in terms of identity work. *Big stories*, the tradition that we started to tackle 10 years ago, are based on the assumption, that we have a life – and we *do* (laughs) I’m not denying that – but that this life is configured in terms of a story. And big story researchers, identity researchers in particular, assume that if we put people into, and let me just be blunt here, when we put them into experimental conditions, almost like taking them to the dark room, putting them on the couch, and letting them forget where and how they really live, and tell them “I want you to reflect on yourself and only yourself”, so “Think about the question, who are you really?”, “Talk to yourself, disregard where you are, what you are, in this particular situation, just dig into your interiority and find out and *lay it all out*, and start as early as you can remember”. This is the kind of confessional, and traditionally an institutional situation. It is the type of therapeutic situation, and this is also a type of the big story research where we typically ask for *life* stories – “You have all the time, two, three hours, and tomorrow we can continue, so tell me everything”. In a way I characterize them as Sunday conditions, you know, appealing to the notion of confessions that happen in catholic churches – I don’t know whether they typically happen on Sundays or not – but that type of experimental condition, within which we are not doing the kind of *normal* story-telling that we typically do, and the assumption behind this type of research, again, is that there is something stored, that is at the essence, that is at the primordial condition of being an existential kind of self that was always there, and maybe changing, but it is part of the story, of *the* story – singular here, that is *you*; or that your *life* is the story that you tell.

I think this is actually *reducing* ourselves to walking stories, to walking narratives. The person and personhood is a walking narrative, and then for us as researchers, in particular in psychology, but in other social sciences as well, we tap into this by letting them tell their story; and this is in no way different from traditional approaches to personality where we use a questionnaire in which we have our participants answer the kinds of questions that *we* think are relevant to this type of identity. So we are stepping outside the normal everyday existence within which stories are told, within which they surface, and assume that there is something that is organizing our life, that we assume is the kind of basic underpinning of being in the world. And I find this highly problematic. If you ask, for instance, 13-year-olds to tell their stories, their life stories, they look at you and say “What are you talking about?” or they say “Well, you know, I was born, and then I went to school” – so they may be able to give you stages. Or if you ask a 70 year old citizen who grew up in the rural parts of China, or has been working all their lives on the conveyer belt, and you go in with a microphone, and say, can you tell me the story of your life, they also will look at you in disbelief and say “What are you talking about? I have been working here all my life, that is me, that’s my life”. So you will not get these book-form life stories with chapters. These are, when you get them as a researcher, *highly stylized forms* that have been exercised and practiced, and this is the way they have come to existence; but they are in no way necessarily *running* – in the sense of determining – someone’s life. They are stylized forms, and that doesn’t mean that they are not interesting or irrelevant. I think they are what they are: highly reflected, retrospective ways of letting one’s life *pass* by oneself, and then drawing some conclusions. So with regard to that, I don’t think they are sitting inside the person. They are ways of making sense, but there are other ways of making sense that are taking place in front of our eyes.

And my call to identity research is simply “Let’s work with ordinary stories first, and then later on you may also want to look at these highly stylized forms that may come in the form of biographies or autobiographies, where there are interesting things to read and to re-reflect on, how these things come to existence. So, I’m not totally doing away with these big stories, these reflective ways of making sense of one self – I don’t want to call them irrelevant; but they are an *outcome* of the identity work in small stories – becoming *formed continuously* in everyday conversations with small stories in their midst. And those stories in there I think are highly interesting, they are really telling, and they also may consolidate in these big stories and these reflective life stories; and as I said, these are forms worthwhile studying, but I don’t understand why – this is a rough estimate – 60 or 70% of research on identity is using *big* stories and not focusing on the *real* formative small stories that are going on in mundane, everyday situations.

**Carolin Demuth:** So, if narrative identity is something that is constructed in everyday interaction, and you contest the notion that there is something like a core inner self that we can get to, are we then not merely rhetorical performers, is there nothing inside of us that is a self or identity? Is it only constructed in the situation?

**Michael Bamberg:** There are maybe two issues here, one is the *interiority-exteriority*, and the other one is the *construction process in situ*. Let me address the first first, because the inside-outside distinction, is one, that is I think is very relevant for psychologists. And traditional psychology I think is build on this notion that we have an inner core, an interiority. Let me tell you a small story maybe, because a couple of years ago, someone asked me to write a chapter for their development of identity handbook, and they wanted me to take the exteriority position, and they thought that this is what I’m doing. And I had this extended argument with them that the distinction between inside and outside is at least a problematic one, maybe even a false one. So at the end I bowed out of it because they wanted to nicely pit my position that identity is something that is formed in interactions in social situations, and not inside a self. So, I said no I can’t do this because that is not what I believe. And then a former student of mine took that position but he also felt it was very misunderstood. So, this contrast of interaction as something that is outside – and there is something that is inside, becomes really even more than problematic, because it is based on a misconception of what language is – what language is, what interaction is. The assumption that seems to run through this is that we have an interiority, wherever that comes from, and sometimes it’s a nativist assumption that is running through this or an evolutionary assumption that is running through this argument, that this interiority then in situ and in vivo is *expressed*. And typically with emotions, right, the emotions are inside, language is placed in the mind or in the brain. So, whenever emotions and language are expressed, they are visible in behavior, but *before* that they are not visible. Nevertheless as psychologists, we can enter what is invisible, what is hidden, the interiority, by having people talk about it, when they unpack it, in particular in talk or in behavior: that’s where the interiority or aspects of it become visible. But, again, this traditional view I think is very close to an experimentalist point of view, that we position the person in experimental conditions, in the dark room out of context, we pull them out of who they are in everyday mundane situations; and only in those special conditions can we find out what is inside. I think this conception is running now through modern psychology in brain research – that we believe we can look into the brain and get answers to what is happening when people are in real everyday contexts. But again, what is running through this is this assumption of this *monologic* self, that we are who we are, when we are with ourselves. When we are in our social contexts, when we are with other people, then we are not our real selves anymore, then we wear masks. Then it is society that *deforms* us. So, this is a way of placing behavior, language in particular, but also interactions, into a context *outside*, that is different from who we really are.

Again, I think what this metaphor between internal and external does is placing the wrong emphasis on – in particular in psychology – on the monologic, the person who seemingly speaks to himself when they are trying to figure things out: the reflective self. And interestingly Hubert Hermans calls this the “dialogical self”. But it is ultimately a very *monologic* way of splitting up the self. And this is only possible because in modernity we have a metaphor giving us the impression that we can find ourselves by going deeper into ourselves, but who is the finder of this finding process, and who is the one that is reflected on? Again, I’m not trying to take this away, I think self reflection is a *very* important achievement in modernity, and this is exactly I think what has become, now part of our modern education processes, that we want all learners become more reflective of the practices in which we are involved; and in order to do that, we have to engage in self-reflection. But the context within which self-reflection makes sense is that we are, first of all, considering the interactive social situations within which we are embedded. Now, we reflect on *those*, and in that process, we also can remove, take a third person perspective so to speak on ourselves, and look at ourselves: what has led to my action. And this again I think is the metaphor that runs deeply through big story research; but it also runs through this whole distinction between interiority and exteriority, and unfortunately it turnes what is called within this metaphoric approach, the exteriority, into a *byproduct*; and story-telling in our everyday interactions into something that also happens – but, you know, it’s not really that relevant or interesting. It is the *interiority* from where all this happens, from where all this comes. This to me is the central point, and this is an outcome of essentializing and existentializing self and identity, from where a lot of these kinds of problematic issues have emerged over the last forty/fifty years with regard to narrative – and over the last hundred, hundred fifty years with regard to psychology.

**Carolin Demuth:** That brings us to the next question. There are narrative identity researchers who refer to self stories as a form of experiencing the world, especially in the realm of phenomenological analysis. What role do phenomenological aspects have in your approach?

**Michael Bamberg:** In my own reflections on narrative and qualitative inquiry, I think this is where the notion of experience has proven to be very important for the narrative turn, and, in the turn to narrative, for making use of narratives. It appears to be the case that stories and experience have a lot in common. Let me step back for a second and get back to the notion of reflection, and enter this issue from there. Because when I tell a story of yesterday as part of an ongoing interaction, so this is a short story of what happened yesterday, and then we continue our conversation. This making something from yesterday relevant to the here and now, the ongoing conversation, is of course also a reflective piece, it is stepping out of the conversation right now and bringing something in from a previous time. So there is some form of reflection, but this is different from sitting down in the dark room or on the couch and reflecting on my whole life. Each small story, each event in which we make something past, possibly even something futurist, fictive, relevant to the here and now of the ongoing conversation, has reflective aspects. In that sense there is an overlap between talking about yesterday or something that happened five years ago, and making it relevant to the here and now and the notion of experience [of what happened]. The term experience has been used, possibly even overused within the narrative turn or the turn to narrative, and I’m also trying to bring this a bit back into what experiences *are* and what they are *not*. So is narrative a form of experience, or can it be? Yes, it can. But behind this is a larger question, a larger bunch of questions.

First of all, let’s just assume for a second, what is it that actually happened, that is made relevant in a story? Then, how is what actually may have happened brought into a sequence of events or happenings that we can call “the experience”, where there is something that you, I, anyone as a subject, subjectively from a particular point of view, have become part of. So, you only could look at what actually happened, an accident, from the street corner or out of the window from where you saw, whatever unfolded, in terms of a sequence of events. So, this brings us to the next distinction, and that is: what when you close the window and move on: what have you stored? which becomes a problematic matter for what is called memory. Now, of course we have to ask how over time this is being reworked through other experiences that were previous, or through new experiences that are similar. So, how has this particular sequence of events that happened, how have they been stored and restored in memory, over time? And then, equally important – or possibly more important: in a particular situation, you bring this up, you make what you think has happened, what has been stored and reworked, you try to make this *relevant*, for a particular interactive situation, like on the couch if you are asked to talk about your mother – and you try to make something relevant from way back to the here and now. And of course, typically this happens in a more interactive situation, because something has been talked about before. and you *know* where the conversation is going, typically, and *that’s* where you bring this in, for a particular reason. So you, first of all, put this into the language that we are habitually speaking.

In Maori, you would ‘language’, as a verb, this particular experience very, very differently, culturally, historically, as you would do this in German or in English – languages that place the subject in sentence initial position. So, with a language already comes also a particular ‘perspectivizing’ of whatever the experience was. But now with the *telling* of it, you are working up particular parts of the sequence of events, you pull them out and plug them into your story. So, in languaging and storying, what you think happened, what you ‘stored’ and what you reworked, comes to existence in the form of a story. And this is different from what you experienced. So, we don’t want to do away with experience, Experience is still, let me carefully say, not irrelevant, but the *telling* of it, let’s focus on that, let’s work with that, let’s work with the micro-cues, that you use in the process of telling, fitting something previous into the here and now and making it relevant, is where we should start. How is it told and why, and those are the kinds of analytical questions that I think we would want to start with; and then we can carefully, if you want to, bring ourselves back to the notion of experience. But what experience nowadays is, has been so wildly problematized, in particular when it comes to traumatic experiences or experiences that are grounded in some larger experiences. Let’s not go into more detail here, but my point is that our belief that we are getting from the stories that people tell to their experiences has become a highly problematized assumption. However, I nevertheless trust that in our analysis of story-telling practices we are much closer to the experiential world of our participants than when we take them out of their everyday context and subject them to answer questionnaire-like questions that either confirm our hypotheses or disconfirm them. And again, I don’t want to say that big story research is a bad approach, but it is not the best approach if you are interested in the experiential world of people and in particular if you look at how they are *dealing* with whatever it is that they think their experiences were. And this would be in the everyday processes of story-telling.

**Carolin Demuth:** Your approach can be broadly located within the field of discursive psychology. Do you see any differences to other approaches within discursive psychology, e.g. approaches that take more a conversational analysis approach, for instance?

**Michael Bamberg:** As I mentioned already, one of the aims of situating stories in our story-telling practices is looking at when people actually tell stories and move from there, what these stories are, what these stories do in the interaction, but also what these stories do in terms of bringing off a sense of who I am that I’m practicing in story-telling – and again, not only in story-telling when I thematize the self but also in story-telling about my children or whatever, where I clearly position myself with my values and my orientations. The original point that I already mentioned was that I think we are more than walking narratives, it is wrong to reduce people to narratives or personhood to narrative. So that is on that extreme. The other extreme would be to say identity doesn’t exist, or it’s an epiphenomenon, and what we are really interested in are just the conversational practices that we engage in, the turn-taking machinery that is keeping interaction going.

I think there is *more*, there’s something, I don’t want to call it in the middle, but I think we can learn from these two extreme positions because in the process of ‘inter-acting’, of being in social situations where we present, display a sense of who we are, we come across, we are affirmed, we react to, this is where we exactly also practice and turn these practices into more and more habits or into rituals of identity work, that become metaphorically speaking ‘us’. So, the way I approach a position that is different from these two extremist positions is by highlighting the story-telling *practices* that we engage in — but not that these practices ultimately result in the one-and-only story that ultimately becomes or replaces me. Rather, we *are* these practices within which we fashion, refashion, rework, navigate, construe a sense of who we are. But also there is this building process and this process of continuous change, that makes it possible to talk about, retrospectively, potential major transformations; but usually there are these small little things in everyday routines that are not always the same, that make who we are, that are forming a sense of who we are, that are forming, or that are answering, or that make it possible that we can answer the question, “Who am I?”. So, I think this is where we can learn in particular from those analysts who work with fine grained situated conversations or interactional data, and more and more also turn to visual data since we have the technical equipment to analyze what is actually going on in these micro-processes in terms of a mutual understanding, in terms of something that some people call the inter-corporeality within which, we as bodies and as minds and brains and as persons, identities, all kind of form or bring ourselves off – I think that’s the best metaphor that I can use at this point in time – a sense of self is brought off and is renewed, but there is a continuous process within which this takes place.

**Carolin Demuth:** There has been quite a bit of discussion and critique within the last few years that qualitative research in general and discursive psychology in particular put a too strong focus on language, and there is a lot of discussion about materiality in which social interaction is embedded in, the material world as well as embodiment. To what extent do you take that into account in your approach?

**Michael Bamberg:** This is a really interesting question. I used to say that without language, well I still do say this [laughs], without language, what we call being human, wouldn’t be the same. Now, that’s easy to say, I think that’s relatively safe. Still, I mean the question here that is behind it, is what is the role of language in having become a language or languaging animal in our genealogy and also in our socio-genesis? How important is language? But I think there are more and more approaches now that try to place language into the body – that we resonate with ourselves when we speak, so to speak – this also then can be pushed into the level of content, and we haven’t gone into that today; the issue of narrative as form and narrative as content, and *using* narrative in order to make sense of form and content and ourselves – but where the body I think is becoming more of an analytic object. And the body has been disregarded, disrespected maybe even, in psychology, and the tradition of psychology, because traditionally it was the soul and then the mind that formed the centerpiece of being human. The body again was more the outside, keeping the inside, in particular the mind and our secrets, all hidden. Now, I think, and maybe that is erroneous, that the change in putting more emphasis on what we do with our bodies, has come from technology, that we see bodies more as – in interaction – as *doing* identity work, as presenting a sense of self and we also have more access to intercultural, visual images these days.

So, what we can do now is break down and display bodies in interaction in slow motion, or in fast forward. We can stop the tape, rewind it, go over it again; and having done this in the last twenty years more and more and with better precision is almost like what in the sciences was the magnifying glass or ways of looking at things that were invisible to the human eye before. Now we have behavior, bodily behavior – and within this, language behavior, under the microscope, under a different microscope; but we can look at the micro-cues that are being, in a way, exchanged when people are doing social work, and in this social work, do identity work. I mean presenting a sense of who we are and hearing and seeing who the other is, and making sense of this as something that works together toward a mutual understanding. So, this is I think where bodies have become much more and much easier of a target of recent analytic frameworks within which language is put into a new frame of doing linguistic analysis – not anymore from written texts, I think this is really past, and in particular written text working with fiction. This is where a lot of identity work, I think, originally in narratology was housed, then to more and more linguistics, where tape recordings and transcripts were the way we tried to fixate spoken language, into sentences, into narratives, and now to the kinds of ways within which language is used, and narratives as well, in bodily work that is taking place in everyday situations but also I think in situations where people are in “life-story-couch-situations” and reflect on who they think they really are.

**Carolin Demuth:** You still refer to language as the main tool to tell stories. What about people who don’t have language? What about deaf people? Are they not able to construct their narrative identity?

**Michael Bamberg:** This brings up exactly the sense-making in deaf and blind populations when it comes to the body. How do blind people make sense of bodily behavior, of gesture and eye gaze, things like this? These are really interesting questions. But behind this also is the question whether there are universal approaches to identity, or whether there is a way – and can narrative identity formation processes still be used as an analytic tool? – to get into sense-making of self, sense-making of others in a more culture-specific way. And in terms of traditional ways within which we are tied into communities of sense-making, within which these bodily, linguistic practices take place, I think that’s where, as long as we can participate in these practices, we are participating in meaning-making processes within which bodies, within which minds, within which habits are forming the foundation for mutual understanding. As long as the aim of mutual understanding, that is tying people together, and that is tied into the practices, into concrete practices that blind people, deaf people, people who can’t speak are tied into, there is a participatory framework within which aspects of this skill set are being practiced. So, it is not the language per se, it is not hearing, speaking per se, or hearing per se or touching per se, I think touching is a very important other skill set in corporeality, in mutual sense making. And then also, laying eyes onto objects and also facial expressions, of smiling, of expressing anger or distance. All these are not necessarily coming from inborn reactions, they might, but that’s not where they are meaningful in our developed, human societal work that we do with each other. This is where the social layers have become the ones that are helping us to interpret the other and self, world – and then my place within world. I think as long as there is a participation, there is a way of learning and being able to practically use those means that are available to the individual, culturally and also in terms of the abilities, the skill sets, that individuals have, and sometimes don’t share.

**Carolin Demuth:** A related question to this: you already mentioned cultural differences, people like Charles Taylor have argued that self narratives have become particularly prominent, you said this yourself earlier in the interview, that self narratives have become more central in our post-modern Western world. Would you say one can take your approach, methodologically, and go to a different cultural context and do research, e.g. in traditional societies where in everyday interactions, self-narratives don’t play such a central role?

**Michael Bamberg:** I think I know where you are heading with this. Cross-culturally, I think to take the self-reflective narrative into communities that are, if there are any, that are not touched by globalization, by Hollywood, I think would be highly problematic. I alluded to it when I said: ask a thirteen year old for a life story, or go ask Chinese workers who are coming from the countryside and now spend twelve to fifteen hours at the conveyer belt, for their life stories. You don’t get what you get in a late modern society from middle class participants. In particular, I think this is where a lot of narrative research moves into the narrative of doctors, of teachers, of nurses, in professional organizations. And there’s nothing wrong with that; but I think that’s very different to use the big story, life story narrative approach in these communities compared to the thirteen year old or non middle class rural, culturally differently designed spaces. BUT, the narrative practice approach, I think still holds water here cross-culturally, as well as *culturally*, because this is truly a cultural approach: you go into their practices within which references to past events or future events are surfacing. This is where something is made relevant that happened, supposedly previously to the interactive context. These are situations, where broadly speaking, the dilemma of *past*, *change*, *present*, *constancy*, so what do past events have in common with the present right now, and what is *different*, are *managed*, are *navigated*.

Right, and that is my argument in the story-telling practices approach: when it comes to the navigation of *differences* between characters, narrative is a good place to look for this type of navigation. When it comes to the navigation of *agency* – how are characters placed, highly agentive, transitive, having an effect on the world, or less so as being affected by the world, in particular in suffering, victimized narratives – again, narratives are a good place to spot these kinds of navigations. But, the navigation of sameness and differences between people, and agency also can be navigated in other genres. But when it comes to the navigation of *past*, *present*, or *different times in the past*, where people are trying to make relevant *changes,* or they make relevant the *constancy* – I’m exactly the same nice young boy I was when I was five years old – where that is the attempt to accomplish – this type of navigation – that’s when narratives are doing, and *only* narratives, this type of job. And it is exactly because they have a temporal dimension. Now recipes and route descriptions also have a temporal dimensions, but not in the same way as narratives. This is simply where you follow a list, so to speak, but narratives are more than a list of events, and we can go deeper into that but I think we past that stage in the interview. I think, this type of narrative practice approach is particularly relevant for what people call ‘cultural research’, what I call ‘contextual research:’ context within different cultures, even in different times, in different situations, in different populations. I think this is more productive than the big story approach that argues that we have to find people’s interiority and unfold it, and show who they *really* are, because small story research fosters understanding in the exemplary fashion. Again, big story research is *not wrong*, and we don’t want to do away with it; but I think there is this whole other side which probably is more complex but also more interesting; and I also think ultimately it will pay off: it will lead to deeper insights. And I think it is typically in sync with qualitative inquiry. So this is where narrative inquiry, the way I define it, is a nice complementation, and fits in a qualitative interesting way.

**Carolin Demuth:** Now you also teach qualitative methods related to your approach. In the last ten or twenty years, various attempts have been made to define criteria to evaluate the quality of qualitative research. It is of course difficult [to define general criteria] because qualitative inquiry is a very heterogeneous field and there are no standardized procedures, but some attempts have been made. But particularly within narrative inquiry, and there are various approaches here as well, it seems that there are no clear-cut criteria of how to evaluate research. Are there any criteria, or do you have any ideas of how we can evaluate the quality of narrative inquiry?

**Michael Bamberg:** This is a really complex issue right now. There are two aspects, one, how does narrative inquiry fit qualitative inquiry, and the other, what really should be called ‘qualitative’, what should be called ‘narrative inquiry’ and possibly what isn’t. These are also kind of political issues, I mean, nobody owns the term inquiry. There is a journal called *Critical Inquiry*, I think that’s the most famous one, then there’s a journal called *Qualitative Inquiry*, and that is pretty well known as well, and there is this journal called *Narrative Inquiry*. So, inquiry is nothing that anyone owns; but what *is* inquiry really and what kind of analytic approach is going along with inquiry? Is story-telling by itself already a type of inquiry? I’m hesitant to follow this, and into this also falls the debate of how much auto-ethnography or whether novel-writing is a form of inquiry. It *can* be argued, but I think this would form a different type of inquiry. It is also a self-inquiry, that’s another topic, it’s an interesting topic and I would love to have that debate at some other point in more detail.

Turning to *narrative* inquiry: what is narrative inquiry? Briefly, the traditional narratology is squarely narrative inquiry: trying to figure out what is a narrative, what are the forms of narrative, how have they historically developed, and also what are the *contents* that typically are put into narratives, the history of the genres and of the themes that have been narrativized. I think those topics are part of literary analysis and literal history and they have formed the meat of narratology in the past; and they are a form of narrative inquiry. And these are also not just embraced in literature or foreign language departments, but also in other departments where people in text linguistics or cognitive linguistics and in cognitive psychology have tried to figure out how certain forms, certain sequences of narrative chunks affect recipients’ minds. Now when it came to taking narrative inquiry as the metaphor to explore lives, and people’s experience, as in the *social sciences*, I think this was a game changer, triggering the big change for the turn to narrative in the social sciences. The realization that we can have people tell stories and we find out how they organize whatever it is – their own experience, their personal lives – and we also use their stories to find out about their understanding of the institution, within which they work, the classroom within which they operate, the interplay between classroom and outside the classroom, the students’ lives so to speak, this realization catapulted narrative inquiry into the public domain. Narrative inquiry became the major tool to use narratives, story-telling as a way of trying to find out or better understand particular segments of private and public lives – how people meet their partners on the web, how they make sense of themselves and others in intercultural social situations.

All this I think contributed to this *huge* wave of adopting narrative into the social sciences as a major tool to investigate, to do inquiry. But that’s also where now a lot of confusion came with this, and this takes us back to the discussion we had before, because some people argue that those segments of life just like the individual personal life are narratively organized. So, some people build narrative into life per se and argue: life is a narrative, that is, narrative is organizing what is happening out there; it is organizing your experience. The next one is, how you *store* your experience, narrative is helping you to store experience, to organize and reorganize experience, to make relevant and then to *narrativize* it. So, some colleagues within the social sciences have placed narrative and these different parts of what we can call now the *narrativization process* to become the central organizing forces for human life. In contrast, and as I try to make clear, I’m trying to locate narratives where people actually break into narratives and tell stories; and I start working from there. Outliers of this position that expands the notion of narrative seems to be holding that *everything* is narrative, and wherever you jump in, you find aspects of a process of narrativization. That’s what I have characterized in my own writings as the “*narrative über Alles*” [“Narrative above all”] position – that I don’t think ultimately holds water. I don’t think it is helpful. I think it leads to a position where anyone can say anything in their analytic work and maintain that they are doing ‘narrative inquiry’. Well, ok, but *how so*? Exemplify it, lay it out, and that’s I think, having used this metaphor of this continuum, from everywhere to actual story-telling practices, that those who venture more to the extreme of essentializing narrative and making it a precondition to human existence, building it into not only human communication but even to *being* human per se, that it leads to a way of shortcutting the kinds of justifications for the questions that researchers are asking and then also for methodological approaches, the tools that they bring in where is it that they actually credit narrative story-telling, to the sense-making abilities that are approached, that are being researched.

So, I haven’t answered your whole question – now bringing this into *qualitative* inquiry, I think, the relationship between qualitative inquiry and narrative inquiry within this range of different perspectives makes it even more problematic. Because qualitative inquiry also is a conglomeration of a number of different ways of asking meaningful research questions and finding ways of answering these meaningful questions. And interestingly, the American Psychological Association (APA) has just put together a task force, and we meet in two weeks actually, for the first time, in order to – and this is an interesting and problematic thing – in order to define guidelines for the publication of qualitative research, and guidelines for the publication of qualitative research obviously also implies there are some guidelines for what *is* good qualitative research, so that it is publishable and should be published. At this point, we are using the guidelines that have been developed and are published in the APA guidelines for qualitative *experimental* research. How much those guidelines are good guidelines that can be transferred to qualitative approaches, is still an open question. But also, some debate will focus heavily on whether there should be *any* guidelines at all, because the argument can be made that we should continue to be open to the kind of innovative practices that have originally turned us to qualitative inquiry, and there I think in particular to narrative inquiry.

One last word, when I teach qualitative inquiry, and I just put together a sequence of thirteen kind of TED talks, small talks, that I want to make available to the public. I’m trying to bring qualitative inquiry into twenty-first century learning strategies. We have changed from, I think largely at least, from the focus on *teaching* practices to *learning* practices and within the learning practices that are replacing lecturing and the transfer of information, qualitative inquiry has become a very powerful instrument to actually show and document how learning practices nowadays can be more productive and more useful for learners in the twenty-first century. And within qualitative inquiry, the way I teach it and the way I have also outlined it in some of these small talks, is that observing is still the queen of qualitative inquiry; so that we have to first of all problematize our everyday modes of laying eyes on others and describing what we see. So, in other words, we have to learn to ask: what is going on when we fashion descriptions in inquiry as researchers, and what kind of interpretative cultural procedures are at work when we describe? It is as simple as that. And from there I think a skill set can be developed within ‘the qualitative mindset’, as we call it, that can be transferred and moved into narrative inquiry. Narrative inquiry also as a highly interpretative discipline where there are interpretative strategies at work that we again have to bring out and then make available for criticism, for reflection. Description, observing, and interpretation of narratives are the pillars, I think, for qualitative inquiry.

Now the question is: how are we able to bring some standards into this that mark and demarcate what is *good* quality from what is *less* quality; I think because we all agree that certain of these ways of doing inquiry are better than others – not everything is the same here – but, we are in highly contested territory, and entering this, I first thought ‘I’m not going to go there’, but then I thought ‘why not?’, this is definitely a terrific learning experience. And at the next Congress of Qualitative Inquiry in Urbana Champaign, this will hopefully be discussed, as one of the core themes. But I hope that we also can bring this back to *narrative* inquiry and *identity* analysis, and I hope that one of the next places we can do this is the International Psychology Conference in Yokohama, in July 2016. So, I think those are places where these issues need to be discussed; and this is an exciting time, and I find things are going in the right direction rather than that there are dangers and problems and issues that we have to resolve. I think it’s an open territory and I think it is fascinating.

**Carolin Demuth:** This brings me to my very last question: What are some future directions that you see for the field of narrative inquiry?

**Michael Bamberg:** I am thinking a lot about that. We possibly can use this question also to divide again a little bit of traditions that are currently on the market so to speak. I think there is one tradition to take narrative out into the fields, partly as a method or as a tool, or something that is inspiring to other disciplines. There is a big discussion in the medical humanities of the role of narrative and even in educating physicians. There is a big discussion of the role of narrative in legal discourse and in legal persuasion; and again, I think in educating lawyers and educating the public also about this. Narrative marketing and narrative branding are, I think, fascinating and I’m trying to get into this as well, in particular with visual narratives: how we can use them in terms of appealing to an audience emotionally but also productively [laughs] not just for consumption to persuade them to consume or identify with a particular brand, but also to become critically involved emotionally, with branding processes and identity processes – *personal branding*.

Right now (November in 2015) we are going in the United States through a process where people are beginning to pick candidates of the two parties who will run against each other next year in the presidential election. And this is where story-telling, I mean it’s just *unbelievably* at the forefront, and it hasn’t been, I think recognized by the media yet. Just a couple of days ago, one of the forerunners of the Republican party has told his story – it is a redemption story – his story of having been a child with a lot of anger problems and having overcome these problems and having become a major figure in a medical discipline. And now presenting a sense of self that is calm, that is comfortable, that is peaceful – so this is an interesting narrative. Now the press, the media has tried to, like with every political candidate, go after this to corroborate the story. And now this life story or this way of trying to come across with this life story as a particular kind of person, has been pulled into doubt. And to me, this is worthwhile a debate again – what are the facts? Or, is it just important to say: this is the way the person who is marketing himself, who is branding himself this way, this is the way the person makes sense of himself, so let it be there and let’s work with what this person has to offer, whether this is a *true* story or *not* a true story, but this is a way he would like to see himself and make sense of himself. This is at the issue right now, of *personal branding* and also in apologizing – something that I have been looking at in the past. I can go on with narratives’ role in education, in marketing, in teacher identity, in all brands of life. So, narrative has become a really *highly* publicized tool of doing this type of inquiry, and that is one direction I think narrative inquiry will continue to become even more popular, more interesting, more applied. I think that is where a number of new journals have come up; it is an exciting area, and I definitely want to be involved in the future, in the personal branding of myself maybe even as well [laughs], but, help people do this, the narrative marketing. So, this is a section of life I think where narrative will expand.

Now, the other – I think that’s also where I would like to say this is where narrative inquiry as a specialized form of inquiry should center – is to reformulate the questions that I also just went over from my own perspective, and this is the discussion of “what *is* narrative?” – This discussion focuses on how narrative is used for identity research in socio-linguistics, in psychology, in sociology and anthropology, and neighboring disciplines. Furthermore, how is it moving from here into these more applied disciplines like narrative marketing and branding? What are the dangers? What are the problems? Should we confine it more? Should we open it up? These are the kinds of discussions that I definitely would like to see more discussed and published within the journal of *Narrative Inquiry.* These are ways of centering on issues that we, I think, as a relatively small community, should also, in a way, control. I mean not control in terms of: this is right, and this is wrong; but enable a discussion within which we find agreements and disagreements, and can widen it – but not too wide – into those applied areas, because the applied areas all provide many ways of publishing applications of narrative inquiry in these fields. So, there are lots of journals, even journals that have narrative in their title, but narrative inquiry itself, I think, is where we might want to provide a forum to center on the discussions of how we work with narrative. What is good work? What is productive work? What is helpful for disciplines? And also what is helpful for expanding the term narrative – or narrowing it in particular ways? And this is, I think, where discussions could take place that we did not get into today: what are differences between ‘identity’, ‘narrative identity’ and ‘forming a narrative sense of self’? How is the question of ‘who am I?’ answered by use of different narrative approaches? This is an area of narrative inquiry, that I would also typically invest myself in the next five or ten years, and I think it’s a growing debate here, and also potentially a very productive one.

**Carolin Demuth:** Narrative inquiry remains a fascinating field and there is still lot of potential for very interesting future research. Thank you very much, Michael, for taking the time for this interview.

**Michael Bamberg:** You are most welcome.
